# Tunnelling current-voltage characteristics of Angstrom gaps measured with terahertz time-domain spectroscopy

**DOI:** 10.1038/srep29103

**Published:** 2016-06-30

**Authors:** Joon-Yeon Kim, Bong Joo Kang, Young-Mi Bahk, Yong Seung Kim, Joohyun Park, Won Tae Kim, Jiyeah Rhie, Sanghoon Han, Hyeongtag Jeon, Cheol-Hwan Park, Fabian Rotermund, Dai-Sik Kim

**Affiliations:** 1 Department of Physics and Astronomy and Center for Atom Scale Electromagnetism, Seoul National University, Seoul 08826, Korea; 2 Department of Physics and Department of Energy Systems Research, Ajou University, Suwon 16499, Korea; 3 Graphene Research Institute and Department of Physics, Sejong University, Seoul 05006, Korea; 4 Department of Nanoscale Semiconductor Engineering and Hanyang University, Seoul 04763, Korea; 5 Division of Materials Science and Engineering, Hanyang University, Seoul 04763, Korea; 6 Department of Physics and Astronomy and Center for Theoretical Physics, Seoul National University, Seoul 08826, Korea

## Abstract

Quantum tunnelling becomes inevitable as gap dimensions in metal structures approach the atomic length scale, and light passing through these gaps can be used to examine the quantum processes at optical frequencies. Here, we report on the measurement of the tunnelling current through a 3-Å-wide metal-graphene-metal gap using terahertz time-domain spectroscopy. By analysing the waveforms of the incident and transmitted terahertz pulses, we obtain the tunnelling resistivity and the time evolution of the induced current and electric fields in the gap and show that the ratio of the applied voltage to the tunnelling current is constant, i.e., the gap shows ohmic behaviour for the strength of the incident electric field up to 30 kV/cm. We further show that our method can be extended and applied to different types of nanogap tunnel junctions using suitable equivalent *RLC* circuits for the corresponding structures by taking an array of ring-shaped nanoslots as an example.

Recently, the terahertz frequency range has facilitated the study of interesting quantum mechanical phenomena. A few examples include electron transport in quantum dots and single molecules^1^,^2^, tunnelling through angstrom- or nanometre-sized gaps^3^,^4^, and Bloch oscillations in bulk solids^5^. Such investigations became possible with the progress of high-field terahertz sources^6^,^7^,^8^,^9^ or with sub-nanometre gaps of metal structures that can probe within atomic distances^2^,^3^. Single-cycle terahertz pulses are suitable for observing ultrafast dynamics in light-matter interactions, whereas the very high field enhancements from plasmonic devices in this frequency range reduce the required intensity of the incident light pulses that would otherwise lead to optical damage. Additionally, with the established technique of electro-optic sampling^10^,^11^, which provides access to both the amplitude and phase of the oscillating fields, it is promising that the terahertz spectral range will continue to reveal novel quantum processes. In this paper, we developed a method to examine the instantaneous current in a tunnel junction based on the aforementioned strengths in terahertz spectroscopy. The tunnelling resistance through a metal-graphene-metal structure was measured, and we suggest that the method can be applied to nano-sized gaps with terahertz resonances.

## Results and Discussion

**Figure 1:**
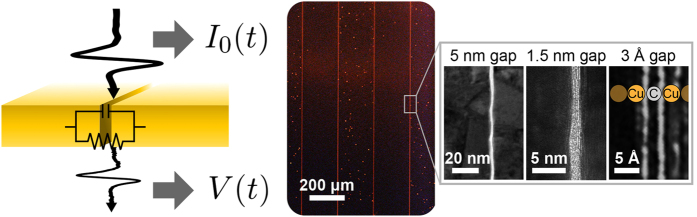
Illustration of terahertz transmission through a slit, represented as an impedance measurement with a current source, which is provided by the incident terahertz pulse. A dark-field optical microscope image and transmission electron micrographs of the nano- and angstrom-gap slits are shown on the right.

A tunnel junction is formed when a vacuum gap or a thin insulating layer is introduced between two metals. This configuration is identical to a slit if the two metals extend far and are optically thick enough to let in the light only through the thin gap, as illustrated in Fig. 1. Here, the fields of the incident and transmitted light coincide with the applied current and voltage that are studied in impedance measurements. When an electromagnetic wave has its magnetic field aligned parallel to the slit (TM polarization), a current *I*_0_(*t*) is induced in the metal, which flows towards the gap. For normally incident plane waves, the current is effectively expressed in terms of a surface current density 
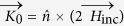
, where 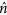 is the unit vector normal to the metal surface and 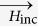 is the magnetic field vector of the incident wave. By contrast, the voltage across the gap *V*(*t*) simply equals the width of the gap times the (enhanced) electric field strength inside the gap. As derived from the Kirchhoff integral formalism, the near-field enhancement is directly proportional to the transmitted field determined in the far field^12^. In short, evaluating the incident and transmitted fields allows for a direct measure of the applied current and induced voltage at the gap, respectively.

For a narrow slit, whose width is much narrower than the metal thickness, a capacitor-like behaviour is expected from the nanogap^13^,^14^. Then, the voltage across gap *V*(*t*) is connected to the applied current *I*_0_(*t*) by the relation 
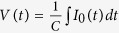
, where *C* is the capacitance of the nanogap structure. In particular, if we consider a harmonic time dependence, given by *e*^*−iωt*^, the equation is rewritten as 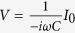 in the frequency domain. To verify that this expression correctly describes the relationship between the incident and transmitted fields of light, we perform terahertz time-domain spectroscopy using an 80 MHz Ti:sapphire laser to generate terahertz pulses from a photoconductive antenna and to detect the pulses via electro-optic sampling with a ZnTe crystal. The following two different metal (gold)-alumina-metal (gold) slit samples are fabricated by applying photolithography and atomic layer deposition (ALD)^15^,^16^: a 1.5-nm-gap sample, which has an array of slits with a width *w* of 1.5 nm, a thickness *h* of 140 nm, and an inter-slit distance *d* of 200 μm, and a 5-nm-gap sample with *w* = 5 nm, *h* = 50 nm, and *d* = 200 μm. A higher signal-to-noise ratio in *V*(*t*) can be achieved with an array of slits than with a single slit, and an inter-slit distance of 200 μm is sufficiently long to neglect coupling effects between neighbouring slits.

**Figure 2:**
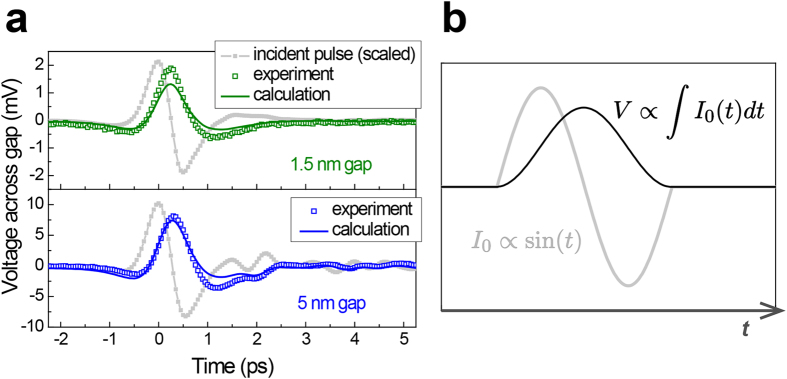
(**a**) Time trace of incident and transmitted terahertz pulses through a slit with a gap of 1.5 nm or 5 nm. Field enhancement factors and gap sizes are multiplied by the incident electric field strength to estimate the voltage across the gap. **(b)** Phase relation between the applied current *I*_0_(*t*) and induced voltage *V*(*t*) of a capacitor.

Using the Kirchhoff integral formalism, the field strength at the near field inside the gap amounts to 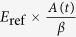
12, where *A* (=*E*_sam_(*t*)*/E*_ref_) is the transmitted amplitude through the sample, normalized by the peak amplitude *E*_ref_ of the transmitted wave through the reference substrate without the slit array, and *β* is the ratio of the total gap area to the total sample area illuminated by the terahertz beam, which is simply *w/d*, by taking into account the periodic arrangement of slits. Thus, the voltage across the gap *V*(*t*) of the slit becomes 
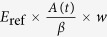
, where Fresnel reflection losses at the front and back side of the quartz substrate, having an index of refraction of *n* = 2.1, yield 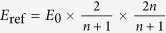, and the electric field strength of the incident beam from the substrate side to the slit array is 
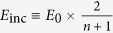
, for which a peak value of *E*_0_ = 20 V/cm is estimated in free space regarding the magnitude of the electro-optic detection signal^17^. Accordingly, THz transmission through the 1.5 nm and 5 nm slits is shown in Fig. 2(a). An aluminium plate with a square aperture, having a side length *l* of 2 mm, which is smaller than the spot size of the terahertz beam, is used to unambiguously define the area of transmission.

Working with the capacitor impedance of the nanogap, the time-dependent transmitted field *V*(*t*) is immediately calculated from the amplitude profile of the incident pulse. The magnitudes of the incident electromagnetic fields are related according to the equation *H*_inc_ = *nE*_inc_*/Z*_0_, where *Z*_0_ is the impedance of the free space. Consequently, a surface current density of 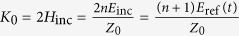 and an applied current of 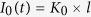 are obtained. The voltage-current relationship from a capacitance *C* = *ε*_0_*ε*_gap_*hl/w*, where *ε*_0_ is the vacuum permittivity and *ε*_gap_ is the relative permittivity of the gap material, finally yields the calculated voltage across the gap *V*(*t*), which is expressed as solid lines in Fig. 2(a). Relative permittivities of *ε*_gap_ = 1.7^2^ and *ε*_gap_ = 2.1^2^ are assigned to slits with gap widths of 1.5 nm and 5 nm, respectively^4^,^15^. Our simple circuit model provides a quantitative agreement with the experimental results without any fitting parameters but by only considering the amplitude profile of the incident pulse and the dimensions *h, w*, and *l* of the capacitor, which are known prior to the measurements. The capacitor picture also explains that the transmitted pulse *V*(*t*) is a consequence of the charge 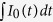 building up and falling off at the metal edges, driven by the single-cycle incident pulse *I*_0_(*t*). In other words, the induced voltage lags the applied current by a phase angle of π/2, as depicted in Fig. 2(b).

As the width of the slit is reduced to the extent that the separation distance between the metals is only a few angstroms, quantum effects cannot be ignored and the optical properties of the gap medium should be reconsidered^18^,^19^. A one-atom-thick graphene sheet is used as a spacer between the two metals to act as a fine slit for terahertz waves, and a substantial decrease in transmittance is observed due to tunnelling electrons^3^. We prepared an identical metal (copper)-graphene-metal (copper) slit sample, and transmission measurements were performed using high-power terahertz pulses generated by optical rectification of laser pulses from a 1 kHz Ti:sapphire regenerative amplifier in a lithium niobate crystal using the pulse-front-tilting scheme^20^. A better estimation of the electric field strength and the voltage across the gap is possible for a high-power terahertz pulse because the estimation from the electro-optic signal can be cross-checked using the average power measured from a pyroelectric detector.

**Figure 3:**
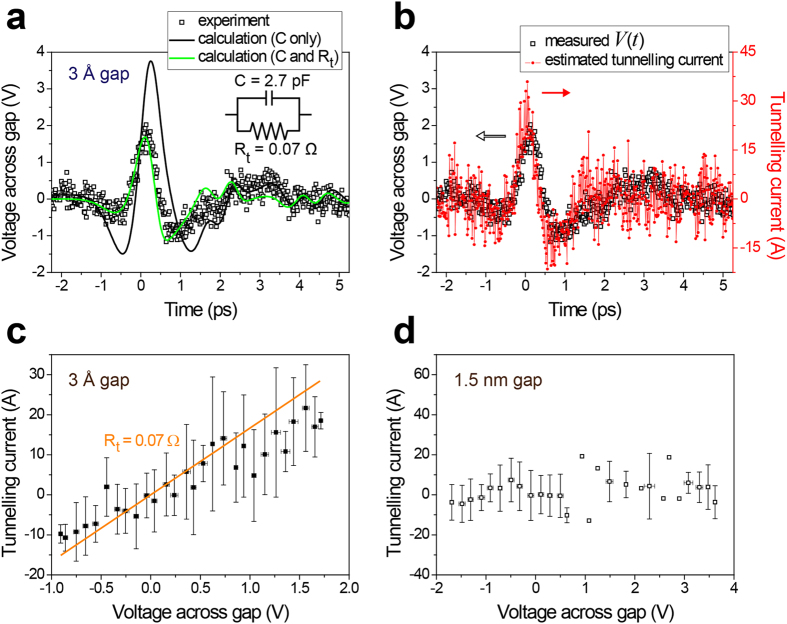
(**a**) Transmission of a terahertz pulse through the 3 Å gap of a metal-graphene-metal slit. The inset shows the equivalent circuit of the gap. **(b)** Measured voltage across the gap *V*(*t*), which is identical to the experimental data in panel (**a**), and the extracted tunnelling current. **(c)** An I-V plot obtained from the results of panel (**b**), and a line that represents a tunnelling resistance *R*_*t*_ of 0.07 Ω. **(d)** An I-V plot of a 1.5-nm-gap metal-alumina-metal slit obtained in the same manner.

For a peak value of *E*_0_ = 30 kV/cm in free space, a maximum of 2 V is induced across the atomic gap, as indicated in Fig. 3(a). The voltage range covered by the terahertz pulse is comparable to that in the dc conductivity study on graphene-metal interfaces^21^. Determined from the structural dimensions of *w* = 3 Å, *h* = 15 nm, and *l* = 2 mm and an effective permittivity *ε*_gap_ of three^3^, a capacitance of *C* = 2.7 pF alone fails to correctly describe the features of the transmitted terahertz wave in this case. Electron tunnelling through the gap prevents charge accumulation at the metal edges and causes a reduced voltage across the gap, and the shape of the transmitted pulse closely resembles the profile of the single-cycle incident pulse. Thus, a finite tunnelling resistance *R*_*t*_ connected in parallel to the innate slit capacitance should be added to the total impedance, which relates the transient voltage to the current applied by the incident radiation^4^. If a constant tunnelling resistance is assumed over the studied voltage range, a value of 0.07 Ω offers a good fit to the experimental results.

Fortunately, the circuit model is able to provide a differential equation that links the measured applied current *I*_0_(*t*) and induced voltage *V*(*t*), and the tunnelling resistance can be deduced without assuming that the resistance value is fixed. The tunnelling current 
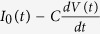
 is obtained from the source current *I*_0_(*t*) and measured voltage across the gap *V*(*t*). The extracted tunnelling current shown in Fig. 3(b) is proportional to the voltage across the gap *V*(*t*), suggesting an ohmic response of the tunnelling electrons through the gap. An unweighted sliding-average smooth of 11 data points is applied to the experimental data presented in Fig. 3(b) to produce a less noisy temporal amplitude profile of the tunnelling current.

Each of the voltage and tunnelling current values at the same instant of time in Fig. 3(b) then yields a data point for a current-voltage plot. Figure 3(c) is a current-voltage characteristic obtained from the mean value of the data points within every 0.1 V interval of the voltage across the gap; error bars indicate the standard deviation. The plot agrees with the slope of 0.07 Ω that was evaluated from the fit in Fig. 3(a). If the peak tunnelling current of ~30 A is normalized by the area specified by *h* = 15 nm and *l* = 2 mm, a peak tunnelling current density of 10^8^ A/cm^2^ is estimated that corresponds to a resistivity of ~2 × 10^−8^ Ωcm^2^. The measured resistivity of an order of magnitude as small as 10^−8^ Ωcm^2^ is close to the theoretical limit, which has been more or less elusive experimentally because of factors related to the quality of the graphene-metal interface^21^.

An alternating current from a high-frequency terahertz pulse is able to probe the overall change of the charge stored by the capacitance of the metal-graphene-metal structure in a contactless manner, whereas conventional dc electrical measurements are likely to be susceptible to local irregularities at the tunnelling interface; the reported values of the resistance from previous dc measurements range from 10^−3^ to 10^−8^ Ωcm^2^ 21,22. If the same process is carried out with the 1.5-nm-gap sample prepared earlier, zero tunnelling current is obtained, as shown in Fig. 3(d), which is reasonable for large barrier widths at moderate field strengths. Our method has a huge room for improvement in the near future because it can benefit from atomic-scale slits using (virtually) infinitely-many kinds of two-dimensional materials, stronger electric fields, and a frequency range extending to the infrared regime.

Because the tunnelling resistance and the capacitance *C* of the slit form a parallel circuit, the resistance being measured should be comparable to the impedance 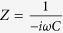 or smaller to be detectable. Therefore, the factor 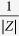 specifies the resolution of our method when measuring the tunnelling current. Smaller tunnelling currents are measurable if the slit capacitance is decreased; presumably, the most efficient way to achieve this in our structure is to shorten the slit length *l* so that the slit reduces to a close-ended slot. Additionally, transmission of electromagnetic waves becomes challenging, as extremely narrow gaps are required to observe quantum effects. Resonance structures, such as slot antennas, allow an increase in transmittance for certain bandwidths, which can also be improved for broadband purposes using multi-resonance structures^23^, and exhibit a better signal-to-noise ratio than slits that suffer from a capacitor-like broad 1/*f*-type spectral response.

**Figure 4:**
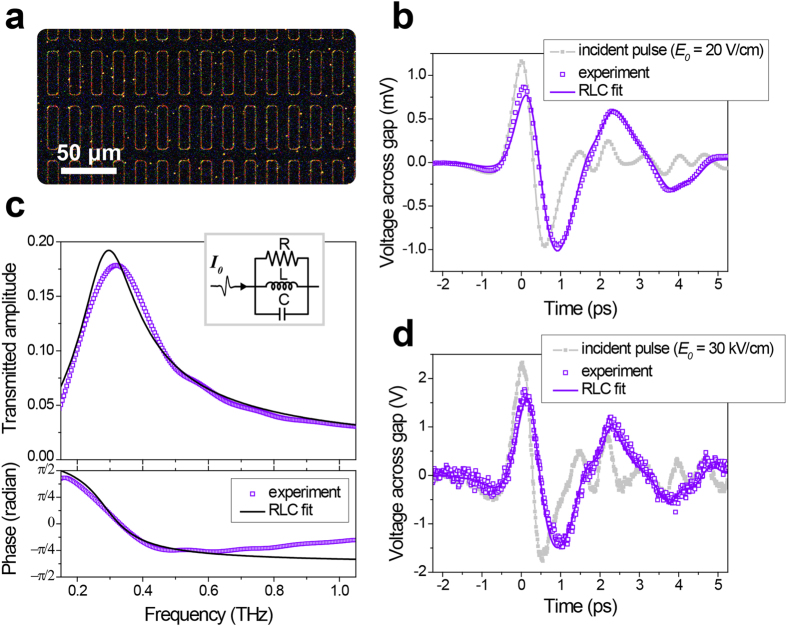
(**a**) A dark-field optical microscope image of the 1.5-nm-gap ring-shaped nanoslot array. **(b**,**c)** Temporal profile of the voltage across the gap and the corresponding Fourier-transformed amplitude and phase of terahertz waves transmitted through the nanoslot array at an incident field strength of *E*_0_ = 20 V/cm. The inset in panel (**c**) shows the equivalent circuit of the nanoslot array. **(d)** Transmission through the same sample by a terahertz pulse with a field strength of *E*_0_ = 30 kV/cm.

For proof-of-concept purposes, we fabricated an array of ring-shaped nanoslot antennas with a gap width of 1.5 nm, as presented in Fig. 4(a). The time trace of the voltage across the gap is shown in Fig. 4(b) for an incident pulse with maximum field strength *E*_0_ = 20 V/cm. The Fourier transformation of the time trace displays a transmitted amplitude as high as 18% at a resonant frequency of 0.3 THz, as shown in Fig. 4(c). In the case of a slot antenna, the equivalent circuit is illustrated as a parallel RLC circuit, as depicted in the inset of Fig. 4(c), where *L* and *C* govern the resonance of the structure and *R* determines the radiation loss or the transmittance through the antenna^24^. The parameters *R, L*, and *C* are determined from the complex impedance for a parallel RLC circuit to acquire the best fit with the transmitted pulse *V*(*t*) from the measured incident pulse *I*_0_(*t*). Moreover, a phase parameter *θ* is introduced to compensate for an additional phase shift in the transmitted wave due to coupling effects between nearby antennas, which is predicted from calculations based on modal expansion^25^. Accordingly, an equivalent waveform is reproduced in Fig. 4(b) by using the equation 
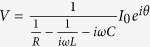
, together with the values of *R* = 5.83 Ω, *L* = 1.72 × 10^−12^ H, *C* = 1.69 × 10^−13^ F, and *θ* = 20.6°. These four parameters are essential to fit the amplitude of the resonance peak, the resonant frequency, the linewidth, and the phase in the frequency domain.

The same procedure is carried out for an incident pulse of *E*_*0*_ = 30 kV/cm. For such moderate intensities, where quantum effects are negligible, the same values of the four parameters also thoroughly duplicate the experimental data, as shown in Fig. 4(d). By contrast, if the measured time trace deviates from the expected waveform, calculated using the previously obtained parameters of *R, L, C*, and *θ*, the difference between the measured and expected amplitude will imply a nonlinear effect associated with terahertz transmission. As presented in Fig. 4(b,d), our simple circuit model serves as an excellent platform to describe the transmission of terahertz waves through nanoslot antennas, even for incident field strengths varying over 3 orders of magnitude.

## Conclusion

We investigated the tunnelling current-voltage characteristics of a metal-graphene-metal structure that has an effective barrier width of only 3 Å by measuring terahertz transmission through an atomically thin slit using the standard time-domain spectroscopy technique. A proof-of-concept experiment was also performed on structures exhibiting resonance in the terahertz frequency range. Our method has the potential to provide insight into both the amplitude and phase related to the ultrafast dynamics of electron transport and allows for the study of alternating-current conductivity measurements with instantaneous fields.

## Methods

By using the adhesive-tape-based planarization method^15^, 1.5-nm-gap slit or slot samples were prepared. A quartz substrate was patterned with gold using the standard photolithography technique and coated with a 1.5-nm-thick aluminium oxide (Al_2_O_3_) using remote plasma ALD. Gold was evaporated once again to completely cover the substrate, and excess metal atop the previously patterned gold was removed using a standard adhesive tape. The 5-nm-gap slit sample was prepared by using the wet etching-based planarization method^16^. A double layer of chromium and aluminium was patterned on top of a gold film, which was evaporated onto a quartz substrate. Gold was removed in places where there were no patterns of chromium and aluminium by ion milling. The remaining structure was coated with a 5-nm-thick Al_2_O_3_ layer via ALD, and gold was evaporated to completely cover the exposed part of the substrate. Subsequent wet etching of aluminium and chromium removed any excess metal on top of the gold nanogap slit array. The 3-Å-gap slit sample was made as follows^3^. First, copper structures were patterned using the photolithography technique, and a single layer of graphene was grown on the surface of the patterned copper layer using the chemical vapour deposition (CVD) system. A secondary copper layer was additionally deposited onto the sample to cover the substrate. Finally, adhesive tape was applied to selectively peel off the second copper layer atop the previously patterned copper for planarization.

## Additional Information

**How to cite this article**: Kim, J.-Y. *et al*. Tunnelling current-voltage characteristics of Angstrom gaps measured with terahertz time-domain spectroscopy. *Sci. Rep.* **6**, 29103; doi: 10.1038/srep29103 (2016).
